# Evaluating the Efficacy and Adverse Effects of Clearing Heat and Removing Dampness Method of Traditional Chinese Medicine by Comparison with Western Medicine in Patients with Gout

**DOI:** 10.1155/2018/8591349

**Published:** 2018-11-13

**Authors:** Nan Xiao, Hao Chen, Shi-Yong He, Chong-Xiang Xue, Hua Sui, Jing Chen, Jia-Lin Qu, Li-Na Liang, Lin Zhang

**Affiliations:** ^1^The First Affliated Hospital of Dalian Medical University, Clinical Laboratory of Integrative Medicine, No. 222, Zhongshan Rd, Dalian 116011, China; ^2^Institute of Integrative Medicine, Dalian Medical University, No. 9, South Road of Lvshun, Dalian 116044, China

## Abstract

*Objective. *In China, the method of clearing heat and removing dampness medicine of Chinese traditional medicine has been widely used on gout. However, the clinical effects are various and not summarized systematically.* Methods. *In this study, a large number of randomized controlled clinical trials were reviewed and analyzed and the clinical efficacy and adverse reactions of traditional Chinese medicine with clearing heat and removing dampness effects for the treatment of gout were systematically evaluated. A comprehensive search of databases including pubMed, China National Knowledge Infrastructure (CNKI), China Science and Technology Journal Database, Wanfang Data, and SinoMed was performed.* Results.* There are 69 randomized controlled trials with 5915 sample sizes meeting the criteria in the study. The results of the meta-analysis indicate that the effects of clearing heat and removing dampness medicine were slightly better than western medicine in the treatment of gout based on the following parameters: serum uric acid (standardized mean difference (SMD):-62.14, 95% confidence interval (CI): -78.12 to-46.15), C reactive protein (SMD: -4.21, 95% CI: -6.19 to -2.23), erythrocyte sedimentation rate (SMD: -6.23, 95% CI: -8.39 to-4.06), and overall clinical response (relative risk (RR): 1.11, 95% CI: 1.08 to 1.15) and, in the profile of adverse drug reactions, the clearing heat and removing dampness medicine showed less adverse reactions than traditional Western medicine (RR: 0.18, 95% CI: 0.10 to 0.32).* Conclusions.* Through a systemic evaluation of the clinical efficacy of the clearing heat and removing dampness medicine of traditional Chinese medicine and western medicine on gout, the clearing heat and removing dampness medicine and western medicine possessed similar clinical efficacy, but traditional Chinese medicine treatments are superior to western medicine in controlling adverse reactions.

## 1. Introduction

With the change of lifestyle and dietary factor, gout has been the global burden [1], mainly because of its high incidence in not only elderly people but also younger people [2]. Gout is a crystal deposition disease which results from local uric acid supersaturation as a consequence of systemic uric acid overload, leading to the formation of monosodium urate (MSU) crystals in the around joints, which caused severe pain and had a strong impact on quality of life. The most common clinical manifestation of gout is recurrent attacks of acute arthritis involving one joint at a time [3]; in some cases, joint injury and renal insufficiency will even occur in patient with gout.

In clinical, western medicines including corticosteroids, allopurinol, and NSAIDs are widely used in treating acute attack of gout [4]. But they all possessed various degrees of side-effect such as gastrointestinal tract reaction, tissue and organ damage, and other adverse reactions. In recent years, IL-1R antagonists appeared and was used for the patients who have contraindications to colchicine and nonsteroidal anti-inflammatory drugs and hormones (oral or injection) [5]. However, the mechanism was unclear and the side effect was also not mentioned. Traditional Chinese medicine (TCM) has been used for preventing and treating gout with unique clinical effects since 200AD in China [6]. Clearing heat and removing dampness is a special medical method for treating patients with gout based on the theory of Chinese traditional medicine, which indicated that the pathogenesis of gout disease is closely related to the heat evil and wetness evil attacking [7]. Many randomized controlled trials (RCTs) in clinical published have selected heat and removing dampness method of Traditional Chinese Medicine to treat gout, and most have clearly shown that Traditional Chinese Medicine have achieved positive results in treating gout.

Meta-analysis, which is the statistical synthesis of relative literature to develop evidence-based conclusions, is able to systematically evaluate and summarize the consistency of multiple studies on the same topic [8]. To the best of our knowledge, there is no meta-analysis of the treatment of gout with heat and removing dampness method of Traditional Chinese Medicine. We need to have a clearer understanding of the application of heat and removing dampness method of Traditional Chinese Medicine in the treatment of gout and to evaluate its effectiveness of in the treatment of gout. The efficacy and side effect of clearing heat and removing dampness medicine and western medicine in the treatment of gout were compared using meta-analysis method in this study. The results will lay a foundation for the treatment of gout with clearing heat and removing dampness methods.

## 2. Methods

### 2.1. Experimental Design

The clinical designs in all reports selected in this study were clinical randomized controlled trials (RCTs). Based on the intervention method, the trials were divided into experimental and control groups, oral administration of Chinese herbal decoction and disposition with other methods of traditional Chinese medicine were included in the experimental group, while oral administration of western medicine was regarded as the control group. The publication time was restricted to the period from January 2000 to May 2017, and the journals' languages were restricted in Chinese and English.

### 2.2. Subjects

According to the diagnostic criteria created by the 1977 American College of Rheumatology classification criteria and Guidelines for the diagnosis and treatment of primary gout established by the Chinese Rheumatology Association, etc., all subjects selected in the study were diagnosed with primary gout in the phase of acute arthritis. Subjects with other comorbidities were excluded.

### 2.3. Database Search Strategy

The titles of “Clearing heat and removing dampness” and “hyperuricemia” or “Clearing heat and removing dampness” and “gout” were searched from the databases including PubMed, China National Knowledge Infrastructure (CNKI), China Science and Technology Journal Database, Wanfang Data, and China Biology Medicine disc during the period from January 2000 to May 2017.

### 2.4. Data Analysis

Three investigators who participated in the study extracted data from all publications selected in this study. The information of the first author, the year of publication, the number of cases in the experimental group and the control group, the intervention method, the end point evaluation index, and the Jadad score were included. One investigator did the first data extraction, the second investigator reviewed the literatures and confirmed the results afterwards, and the third investigator participated in the discussion when the disagreement occurred and reached a consensus with the other two finally.

### 2.5. Endpoint Indicators

The evaluations of effective and ineffective were reached artificially based on the indicators of measurement data, such as Serum uric acid (SUA), C-reactive protein (CRP), and erythrocyte sedimentation rate (ESR). And the evaluations of SUA, CRP, and ESR were collected after the period of gout attack. The reducing of blood uric acid, ESR, and CRP and the relieving of the clinical symptoms were regarded as effective. On the contrary, it was ineffective. The respective measurement data of blood uric acid, ESR, and CRP were also regarded as the primary indicators.

### 2.6. Assessment of Methodological Quality

Assessment of methodological quality is based on the validated Jadad scale by two reviewers (XN and CH) and the Jadad scale has three scoring points. The first is if the study was described as randomized, and with detailed descriptions. The second is if the blind method was adopted in the study and with detailed descriptions. And the third is if there was a description of withdrawals and dropouts. A paper reporting could therefore receive a Jadad score from 0 to 7, in which the scores with 1-3 and 4-7 were considered as low and high quality, respectively.

### 2.7. Statistical Methods

The measurement data were evaluated using the mean difference (MD) and 95% confidence interval (CI), or the relative risk (RR) and 95% confidence interval. If the heterogeneity of the study was within the acceptable range (*I*^*2*^≤ 50%), the fixed effect model was used. Otherwise, the random effects model was used. The collected data in clinical research were analyzed by RevMan 5.0 software.

## 3. Results

### 3.1. Selection of Studies

**Figure 1:**
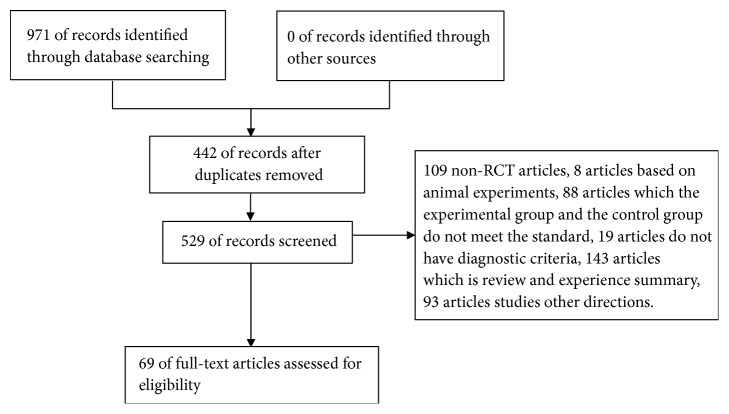
Flow diagram of study selection.

971 articles about the treatment of gout using clearing heat and removing dampness medicine of Chinese traditional medicine were retrieved from five electronic databases, in which 442 duplicated publications articles were excluded. Then two reviewers independently screened the full texts of the remaining 529 articles. 109 non-RCT articles, 8 articles based on animal experiments, 88 articles had inconformity to inclusion standard but included the experimental group or the control group, 19 articles without diagnostic criteria, 143 articles about review and experience summary, and 93 articles about other directions. Finally, 69 articles were included in this study [9–77] (Figure 1). The characteristics of these studies were shown in Tables 1 and 2.

### 3.2. Risk of Bias

**Figure 2:**
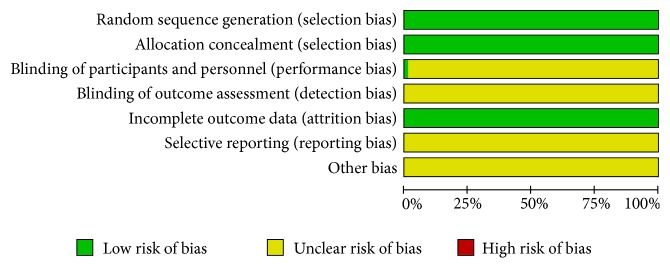
Risk of bias summary.

**Figure 3:**
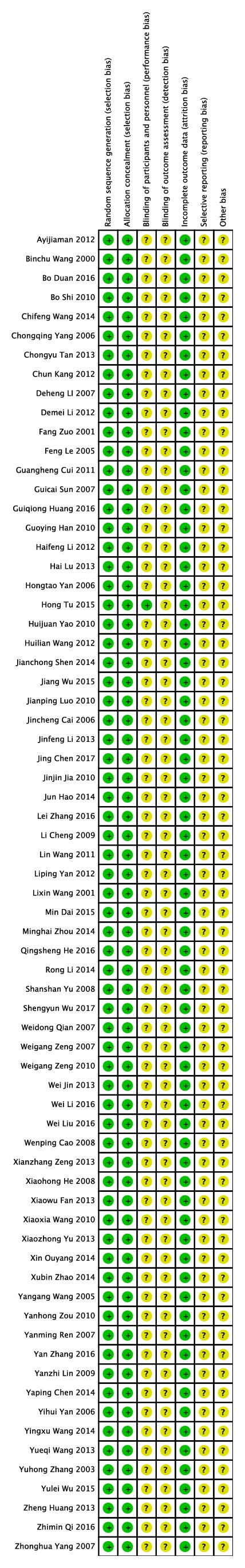
Risk of bias graph.

All of the selected trials adopted the method of randomization [9–77], which involved sealed envelopes11, randomized block [9, 10, 12, 13, 15, 17–33, 36–38, 40–51, 53–77], and random number table [14, 16, 34, 35, 39, 52]. Therefore, those trials were considered low risks in terms of selection bias. Only one trial involved the method of blinding [36]. These parameters were considered low risk in terms of incomplete outcome data. Detection bias, reporting bias, and other potential biases were unclear in all studies (Figures 2 and 3).

### 3.3. Adverse Reactions

**Figure 4:**
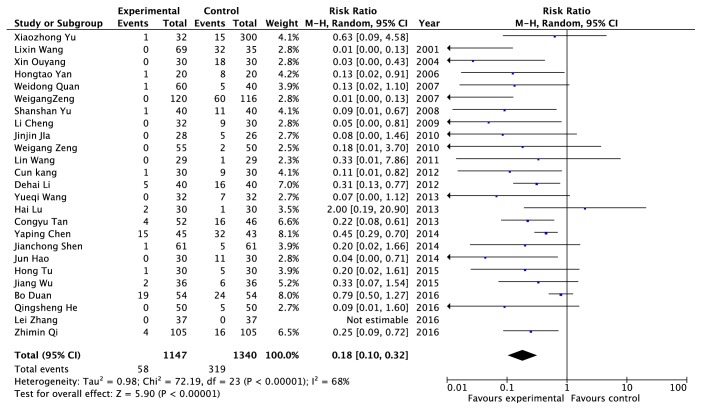
An analysis of the adverse reactions caused by clearing heat and removing dampness method and western medicine in the treatment of gout.

Adverse reactions data were provided from twenty-five RCTs, including 2217 patients (1147 cases in the experimental group and 1070 cases in the control group) [11, 20, 23, 24, 27, 30, 32, 34–36, 38–40, 42, 47, 48, 53–59]. The random model was applied finally because of its heterogenicity with* I*^*2*^ = 68% (Figure 4). The results indicated that the adverse reactions rate of patients taking Chinese herb and (or) receiving other traditional Chinese medicine treatment was lower than that of patients who take western medicine (0.18 times), and the difference was statistically significant (*P *< 0.00001).

### 3.4. Efficacy

**Figure 5:**
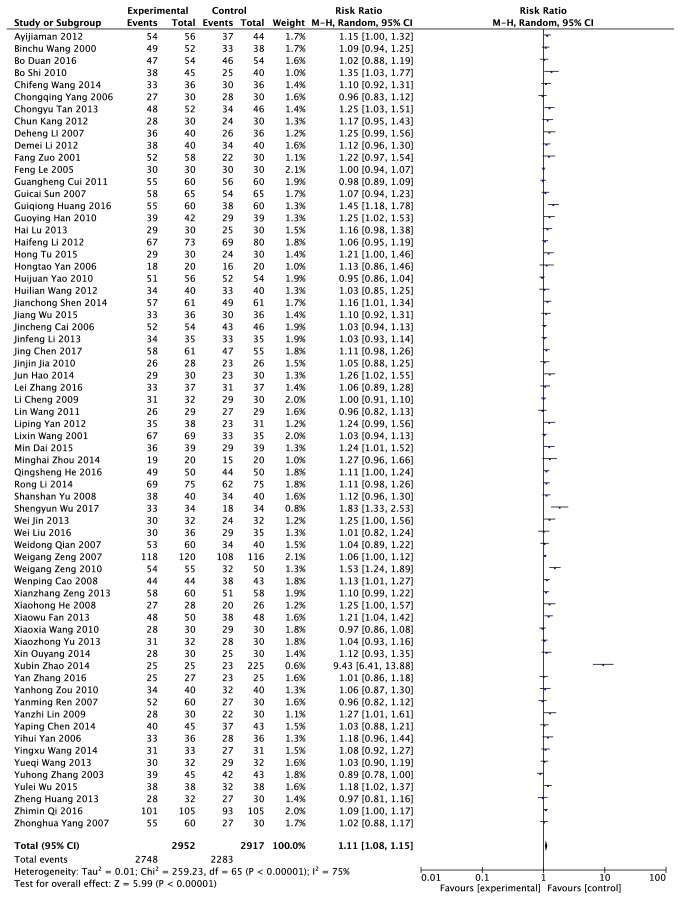
An analysis of the effective of clearing heat and removing dampness method and western medicine in the treatment of gout.

The effective rate data were provided from sixty-six RCTs, including 5669 patients (2952 cases in the experimental group and 2717 cases in the control group) [9–13, 15–44, 46–69, 71–77]. The random model was used because of its heterogeneity with* I*^*2 *^= 75% (Figure 5). The results indicated that the effective rate of patients who took Chinese herb and (or) treated with other traditional Chinese medicine methods were higher than that of those who used Western Medicine (1.11 times). The difference was statistically significant (*P *< 0.00001).

### 3.5. Serum Uric Acid Concentration (*μ*mol/L)

**Figure 6:**
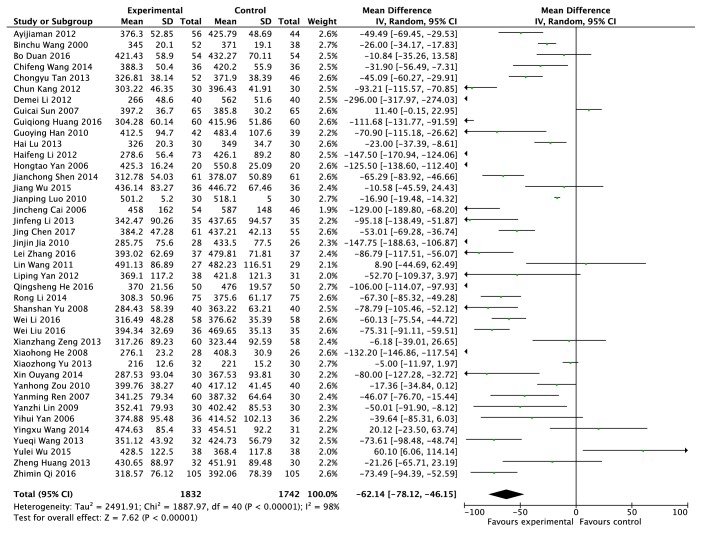
Effects of clearing heat and removing dampness method and western medicine on serum uric acid in the treatment of gout.

The serum uric acid concentration data were provided from forty-one RCTs, including 3549 patients (1834 cases in the experimental group and 1715 cases in the control group) [9–49]. The random model was adopted according to* I*^*2 *^= 98% (Figure 6). Compared with the patients in the control group who only took western medicine, the level of serum uric acid concentration was reduced by 62.14% in patients who took traditional Chinese herb and (or) treated with other traditional Chinese medicine methods. The results were statistically significant (*P *< 0.00001).

### 3.6. C-Reactive Protein (mg/L)

**Figure 7:**
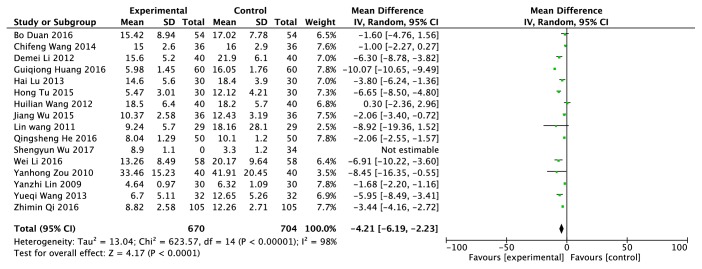
Effects of clearing heat and removing dampness method and western medicine on C-reactive protein in the treatment of gout.

The C-reactive protein data was provided from sixteen RCTs, including 1408 patients (704 cases in the experimental group and 704 cases in the control group) [12, 14, 21, 23, 24, 30, 37–42, 44, 50–52]. A random model was adopted according to* I*^*2 *^= 98% (Figure 7). Compared with the patients in the control group who only took western medicine, the level of C-reactive protein was reduced by 4.21% in patients who took traditional Chinese herb and (or) treated with other traditional Chinese medicine methods. They were statistically significant on the difference of two intervention methods to reduce C-reactive protein levels (* P *<0.0001).

### 3.7. Erythrocyte Sedimentation Rate (ESR) (mm/h)

**Figure 8:**
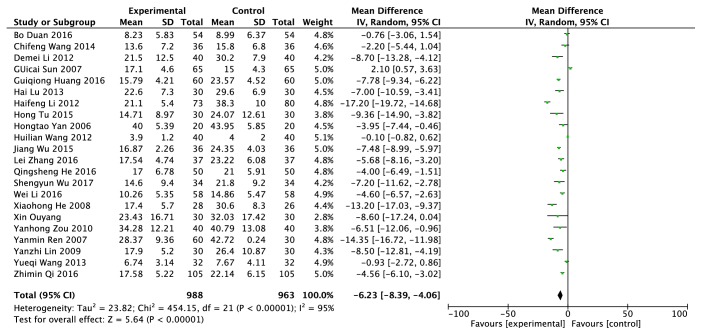
Effects of clearing heat and removing dampness method and western medicine on ESR in the treatment of gout.

The Erythrocyte sedimentation rate data was provided from twenty-two RCTs, including 1951 patients (988 cases in the experimental group and 963 cases in the control group) [10–12, 14, 17, 19, 20, 23, 24, 26, 27, 30, 37–41, 44, 50–52]. A random model was adopted according to* I*^*2*^ = 95% (Figure 8). Compared with the patients in the control group who only took western medicine, the level of Erythrocyte sedimentation rate was reduced by 6.23% in patients who took traditional Chinese herb and (or) treated with other traditional Chinese medicine methods. There was significant difference between the experimental group and the control group on ESR (*P *< 0.00001).

## 4. Discussion

With the continuous improvement of people's living standard, the change of dietary structure and the influence of environmental factors, the incidence of gout has been gradually increased every year all over the world. Nowadays, the patient with gout could only be alleviated but not cured with existing clinical treatments; thus searching for a better therapeutic method has been appeared to be very important. Our current study analyzed data from 69 RCTs that aimed to assess the therapeutic effect and safety of heat and removing dampness method of Traditional Chinese Medicine for gouty arthritis.

In respect of adverse reaction rate, clearing heat and removing dampness method with or without other traditional Chinese medicine therapy are better than western medicine treatment (RR = 0.18). The adverse reactions often occurred during the process of using drugs, such as abdominal pain, diarrhea, vomiting, and inappetence, and even causing damage of liver and kidney in severe cases. Compared with western medicine, clearing heat and removing dampness method was shown to be more effective to the patients with gout, with less adverse reactions mentioned above at the same time, probably due to the synergistic action of multicomponents and multitargets in the traditional Chinese medicine and the integrity of human body. The clearing heat and removing dampness method which is the unique medical method based on Chinese traditional medicine has a good effect to treat patients with gout, mainly because of the pathogenesis of gout disease which is that the meridian is blocked by a pathogenic factor formed by blending of heat and wetness evils [7].

According to our results, the clearing heat and removing dampness method of Chinese traditional medicine adjuvant treatment of Chinese medicine such as acupuncture and cupping could effectively reduce uric acid, C-reactive protein, and ESR in patients with gout. Different researchers have chosen different modalities of the clearing heat and removing dampness method of Chinese traditional medicine, such as Si Miao San [29–31], Xuan Bi Fang [38, 39], acupuncture and Chinese medicine combination [41, 59, 76], etc. Among them, the types of Chinese medicine, the dose, and the acupoint selection of acupuncture and acupuncture techniques are inconsistent. Therefore, our results cannot get a clear conclusion, we do not know which treatment method has the best effect on gout and the least adverse reactions.

In this study, there are many shortcomings: a lot of randomized controlled trials are not amply descripted in grouping; it is not sufficient to prove that the randomization program is executed correctly. The overall methodological quality is poor, which limits the value of the effect of the clearing heat and removing dampness method of Chinese traditional medicine in the treatment of gout. Therefore, an indepth investigation and further extensive study were need in the future.

## Figures and Tables

**Table 1:** Characteristics of included studies.

Author, year	Sample size		Age		Intervention methods		Duration treatment		Effective number		Jadad scale
	EG	CG	EG	CG	EG	CG	EG	CG	EG	CG	
Yan Zhang, 2016	27	25	47.04±12.92	47.72±8.69	Qingrelishi side	Low purine diet + meloxicam	14	14	25	23	3
Fang Zuo, 2001	58	30	N/A	N/A	BaiHuGuiZhi Decoction+ Simiao Powder	allopurinol	14	14	52	22	1
Yihui Yan, 2006	36	36	N/A	N/A	BiNing Decoction	allopurinol	28	28	33	28	1
Xubin Zhao, 2014	25	25	N/A	N/A	Blood-letting puncture and cupping+ Microwave therapy	Colchicine +Diclofenac Sodium Sustained Release Capsules	3~7	1~7	25	23	1
Li Cheng, 2009	32	30	N/A	N/A	Danghuiniantong Decoction	Meloxicam	7	7	31	29	1
Guicai Sun, 2007	65	65	N/A	N/A	Compound xiqiancao capsule	Voltaren+ allopurinol	21	21	58	54	1
Yuhong Zhang, 2003	45	43	N/A	N/A	Modified Sanmiao Powder	Colchicine	21	21	39	42	1
Shengyun Wu, 2017	34	34	35.9±6.1	35.9±6.1	Simiao Powder+ Xinhuang Tablet(external)	Colchicine	14	14	33	18	1
Qingsheng He, 2016	50	50	34.5±4.7	32.7±3.2	Modified Simiao Powder+ Xinhuang Tablet(external)	Colchicine	7	7	49	44	3
Lixin Wang, 2001	69	35	N/A	N/A	Modified Simiao pill	Colchicine	N/A	N/A	67	33	1
Zhonghua Yang, 2007	60	30	N/A	N/A	Jianpi qingrelishi Tongluo Decoction	Colchicine	15	15	55	27	1
Wenping Cao, 2008	44	43	N/A	N/A	JunHu analgesic powder(external)+ acupuncture treatment	colchicine	3	3	44	38	1
Yanhong Zou, 2010	40	40	N/A	N/A	Lizhuodingtong decoction	Nimesulide	10	10	34	32	1
Jincheng Cai, 2006	54	46	N/A	N/A	Niantongxiaofeng prescription	colchicine	15	15	52	43	1
Wei Li, 2016	58	58	N/A	N/A	Qingrechushi prescription	Etoricoxib Tablets	14	14	N/A	N/A	3
Yingxu Wang, 2014	33	31	46.97±9.65	41.16±9.72	Discriminate treatment of Chinese medicine by clearing away heat and resolving turbid	Diclofenac Sodium Sustained Release Tablets+ Benzbromarone Tablets	31	31	31	27	1
Wei Liu, 2016	36	35	46.73±16.25	47.06±15.82	Compound Chinese medicine decoction of Clearing heat-toxin and eliminating dampness method	Diclofenac Sodium Sustained Release Tablets	14	14	30	29	3
Feng Yue, 2005	30	30	N/A	N/A	Clearing heat-toxin and eliminating dampness method+ Gold paste(external)	colchicine	7	7	30	30	1
Yanming Ren, 2007	60	30	N/A	N/A	Acid fat clear capsule	colchicine	7	7	52	27	1
Huilian Wang, 2012	40	40	48.4±12.8	49.1±13.1	Gouty granule	Nimesulide	10	10	34	33	1
Wei Jin, 2013	32	32	C	N/A	Self-prepared gout recipe+ Jiawei Jinhuang powder	colchicine	7	7	30	24	1
Hong Tu, 2015	30	30	44.9±9.1	45.1±8.1	Gout clear granules	Diclofenac Sodium Sustained Release Tablets	7	7	29	24	1
Binchu Wang, 2000	52	38	N/A	N/A	Gout decoction	Ibuprofen+ Probenecid52	10	10	49	33	1
Haifeng Li, 2012	73	80	45.6±10.1	47.2±12.4	Xuanbi decoction	allopurinol	30	30	67	69	1
Hongtao Yan, 2006	20	20	N/A	N/A	Xuanbi decoction	Indomethacin	7	7	18	16	3
Weigang Zeng, 2010	55	50	N/A	N/A	Yushantongfeng decoction 2	allopurinol	7	7	54	32	1
Weigang Zeng, 2007	120	116	N/A	N/A	Yushantongfeng decoction	colchicine	N/A	N/A	118	108	1
Jiang Wu, 2015	36	36	51.34±6.28	52.46±7.4	Acupuncture+Sanren decoction and Sijunzi decoction	Diclofenac Sodium Sustained Release Tablets	7	7	33	30	1
Jing Chen, 2017	61	55	52.1±1.2	51.5±1.4	Chinese medicine and acupuncture	Colchicine+ allopurinol	7	7	58	47	1
Minghai Zhou, 2014	20	20	N/A	N/A	Simiao powder and acupuncture	Ibuprofen Sustained-release Capsules	7	7	19	15	1
Guangheng Cui, 2011	60	60	N/A	N/A	Hoveniaacerbalindl Tongfeng Decoction	Meloxicam	7	7	55	56	1
Yangang Wang, 2005	35	35	45.0±6.5	46.0±5.7	Compound decoction of Chinese herbal medicine	Colchicine+ allopurinol	7	7	N/A	N/A	1
Hai Lu, 2013	30	30	N/A	N/A	Simiao pill and Gouty ointment	Allopurinol+ Sodium Bicarbonate Tablets(oral)+ Qingpeng Paste(external)	7	7	29	25	2
Bo Duan, 2016	54	54	40.26±10.98	42.31±11.77	Oral administration of Chinese medicine decoction+Rebiqing granules(oral)+Chinese medicine(external)	Colchicine Tablets	7	7	47	46	2
Guoying Han, 2010	42	39	N/A	N/A	Rabdosia rubescens, coix seed, Poria cocos, Shi Wei, Rhizoma Atractylodis, Cortex Phellodendri, Achyranthes bidentata, rhubarb, Eupatorium adenophorum	Allopurino+Celecoxib	31	31	39	29	1
Min Dai, 2015	39	39	35±2.5	35±2.5	Atractylodes rhizome, Achyranthes bidentata, Smilax glabra, Cortex Phellodendri, honeysuckle vine, liquorice, 2corydalis tuber, astragalus root and coix seed	Diclofenac Sodium Sustained Release Tablets	14	14	36	29	1
Xiaohong He, 2008	28	26	42.5±7.7	46±11.8	Simiao powder+Sihuang powder	Colchicine	3	3	27	20	1
Xiaoxia Wang, 2010	30	30	48.5±15.5	49.1±13.7	Self-made prescription: Mountain arrowhead, clematis, peach, Atractylodes, Poria, Alisma, Adenophora, Polygonum cuspidatum, rice, Bixie	Colchicine	10	10	28	29	1
Huijuan Yao, 2010	56	54	N/A	N/A	Modified Guizhishaoyaozhimu decoction	Colchicine	10	10	51	52	1
Lei Zhang, 2016	37	37	44.43±11.29	45.02±12.1	Oral administration of Bixie decoction	Diclofenac Sodium Sustained Release Tablets	7	7	33	31	1
Bo Shi, 2010	45	40	49±16	48±14	Clearing heat and dmp elimination tongluo +Relieving stasis and pain	Colchicine	10	10	38	25	1
Liping Yan, 2012	38	31	N/A	N/A	Clearing heat and dmp elimination prescription	Colchicine	7	7	35	23	1
Yulei Wu, 2015	38	38	N/A	N/A	Clearing heat and removing dampness and activating blood circulation method	Sodium Bicarbonate Tablets+ Diclofenac Sodium Sustained Release Tablets	N/A	N/A	38	32	1
Yueqi Wang, 2013	32	32	N/A	N/A	Chinese medicine of Clearing heat and removing dampness and activating blood circulation method	Diclofenac acid enteric coated tablets	7	7	30	29	1
Rong Li, 2014	75	75	50.24±12.37	49.15±13.06	Bixieshenshi decoction and Taohongsiwu decoction	Celecoxib+ Sodium Bicarbonate Tablets	10	10	69	62	1
Shanshan Yu, 2008	40	40	48.2±8.6	46±9.7	Dampness detoxification method	Nimesulide tablets	N/A	N/A	38	34	1
Weidong Qian, 2007	60	40	N/A	N/A	Xitong Granule	Colchicine	7	7	53	34	1
Zheng Huang, 2013	32	30	N/A	N/A	Qingrelishi Tongluo Decoction	Nimesulide tablets	10	10	28	27	1
Chongyu Tan, 2013	52	46	N/A	N/A	Self-made Clearing heat and dmp elimination tongluo prescription	Colchicine+ Fenbid	7	7	48	34	2
Chun Kang, 2012	30	30	70.2±5.3	69.9±6.1	Self-made Clearing heat and dmp elimination tongluo decoction	Ibuprofen Sustained-release Capsules+ Sodium Bicarbonate Tablets	7	7	28	24	2
Xiaowu Fan, 2013	50	48	N/A	N/A	Clearing heat and dmp elimination tongluo decoction	Fenbid	7	7	48	38	2
Jianchong Shen, 2014	61	61	45.78±7.45	46.47±8.35	Laoshitongfeng prescription+acupuncture	Colchicine+ Sodium Bicarbonate Tablets	7	7	57	49	1
Guiqiong Huang, 2016	60	60	53.82±10.95	54.05±12.31	Clearing heat and dmp elimination tongluo prescription	Colchicine	7	7	55	38	1
Xin Ouyang, 2004	30	30	57.77±6.62	56.37±6.74	Bixiehuadu decoction	Butazolidin	14	14	28	25	1
Zhimin Qi, 2016	105	105	45.3±9.2	44.1±9.6	Qingrelishi Zhuyu Decoction	Colchicine+ Dexketoprofen Trometamol Tablets	7	7	101	93	2
Yaping Chen, 2014	45	43	N/A	N/A	Clearing heat and dredging collaterals to clear turbid decoction	Diclofenac Sodium Sustained Release Tablets	14	14	40	37	1
Demei Li, 2012	40	40	N/A	N/A	Clearing heat and dampness, blood stasis, relieving pain Tongluo Decoction; Qingre Tongluo releasing pain prescription	Colchicine+ Celecoxib Capsules+ Votalin(external)	7	7	38	34	1
Yanzhi Lin, 2009	30	30	53.55±10.67	55.73±11.92	Self-made Sanjin decoction and Sanmiao powder	Colchicine+ allopurinol	30	30	28	22	2
Dehe Li, 2007	40	36	44.3±5.2	43.1±4.5	Shandayanhesimiao decoction	Colchicine	14	14	36	26	1
Jun Hao, 2014	30	30	46±8.03	45.16±7.97	Gout mixture	Colchicine	15	15	29	23	1
Chongqing Yang, 2006	30	30	N/A	N/A	Gout prescription	Votalin	7	7	27	28	3
Lin Wang, 2011	29	29	N/A	N/A	Tongbi prescription	Diclofenac Sodium Sustained Release Tablets	7	7	26	27	4
Ayijiaman, 2012	56	44	44.3±4.3	44.9±3.9	Tanrebi decoction	Sodium acetate	14	14	54	37	1
Chifeng Wang, 2014	36	36	51.7±3.2	52.1±2.9	Simiao pill	Meloxicam	N/A	N/A	33	30	1
Jianping Luo, 2010	30	30	N/A	N/A	Modified Simiao Powder	Diclofenac Sodium Enteric-coated Tablets+ Colchicine	3	3	N/A	N/A	1
Xianzhang Zeng, 2013	60	58	N/A	N/A	Modified Simiao Powder	Colchicine+ allopurinol	14	14	58	51	1
Jinjin Jia, 2010	28	26	43±10.8	44±11.3	Modified Simiao Powder	Colchicine	14	14	26	23	1
Xiaozhong Yu, 2013	32	30	N/A	N/A	Simiao powder	Ibuprofen+ Colchicine	14	14	31	28	1
Jinfeng Li, 2013	35	35	N/A	N/A	Simiaomaqian powder	Diclofenac Sodium Sustained Release Tablets	7	7	34	33	1

**Table 2:** Outcome of the meta-analyses for the comparison between clearing heat and removing dampness method of Chinese traditional medicine and western medicine, according to study design.

Author, year	Effective number		SUA		CRP		ESR		Adverse reactions	
	EG	CG	EG	CG	EG	CG	EG	CG	EG	CG
Yan Zhang, 2016	25	23	N/A	N/A	N/A	N/A	N/A	N/A	N/A	N/A
Fang Zuo, 2001	52	22	N/A	N/A	N/A	N/A	N/A	N/A	N/A	N/A
Yihui Yan, 2006	33	28	487.27± 98.88/374.88± 95.48	469.26± 114.41/414.52± 102.13	N/A	N/A	N/A	N/A	N/A	N/A
Xubin Zhao, 2014	25	23	N/A	N/A	N/A	N/A	N/A	N/A	N/A	N/A
Li Cheng, 2009	31	29	N/A	N/A	N/A	N/A	N/A	N/A	0	9
Guicai Sun, 2007	58	54	568.6±21.2/397.2±36.7	572.5±27.6/385.8±30.2	N/A	N/A	59.2±7.4/17.1±4.6	53.7±8.7/15±4.3	N/A	N/A
Yuhong Zhang, 2003	39	42	N/A	N/A	N/A	N/A	N/A	N/A	N/A	N/A
Shengyun Wu, 2017	33	18	N/A	N/A	35.5±3.5/8.9±1.1	35.7±3.3/10.1±1.2	45.7±9.3/14.6±9.4	46.9±9.1/21.8±9.2	N/A	N/A
Qingsheng He, 2016	49	44	511±47.77/370±21.56	497±50.12/476±19.57	31.34±9.12/8.04±1.29	29.77±8.9/9.26±2.55	45±11.26/17±6.78	47±12.57/21±5.91	0	5
Lixin Wang, 2001	67	33	N/A	N/A	N/A	N/A	N/A	N/A	0	32
Zhonghua Yang, 2007	55	27	N/A	N/A	N/A	N/A	N/A	N/A	N/A	N/A
Wenping Cao, 2008	44	38	N/A	N/A	N/A	N/A	N/A	N/A	N/A	N/A
Yanhong Zou, 2010	34	32	442.15±60.12/399.76±38.27	438.58±67.45/417.12±41.45	67.12±30.23/33.46±15.23	60.58±29.40	64.12±25.78/34.28±12.21	60.10±23.69/40.79±13.08	N/A	N/A
Jincheng Cai, 2006	52	43	585±155/458±162	592±142/587±148	N/A	N/A	N/A	N/A	N/A	N/A
Wei Li, 2016	N/A	N/A	536.24±54.16/316.49±48.28	529.75±52.67/376.62±35.39	32.94±5.84/13.26±8.49	33.18±5.63/20.17±9.64	24.21±9.64/10.26±5.35	23.68±9.71/14.86±5.47	N/A	N/A
Yingxu Wang, 2014	31	27	605.21±132/474.63±85.4	601.16±114.2/454.51±92.2	N/A	N/A	N/A	N/A	N/A	N/A
Wei Liu, 2016	30	29	548.25±96.31/394.34±32.69	541.45±48.11/46	N/A	N/A	N/A	N/A	N/A	N/A
Feng Yue, 2005	30	30	N/A	N/A	N/A	N/A	N/A	N/A	N/A	N/A
Yanming Ren, 2007	52	27	544.36±86.27/341.25±79.34	567.74±91.13/387.32±64.64	N/A	N/A	52.61±12.35/28.37±9.36	48.22±11.79/42.72±0.24	N/A	N/A
Huilian Wang, 2012	34	33	N/A	N/A	49.7±11.6/18.5±6.4	47.6±12.6/18.2±5.7	9.0±3.0/3.9±1.2	8.9±2.9/4.0±2.0	N/A	6
Wei Jin, 2013	30	24	N/A	N/A	N/A	N/A	N/A	N/A	N/A	N/A
Hong Tu, 2015	29	24	N/A	N/A	26.28±7.89/5.47±3.01	24.16±6.99/12.12±4.21	39.62±16.51/14.71±8.97	37.74±14.63/24.07±12.61	1	5
Binchu Wang, 2000	49	33	489±23.1/345±20.1	486±21.5/371±19.1	N/A	N/A	N/A	N/A	N/A	N/A
Haifeng Li, 2012	67	69	287.6±56.4	426.1±89.2	N/A	N/A	21.1±5.4	38.3±10.0	N/A	N/A
Hongtao Yan, 2006	18	16	537.85±39.72/425.3±16.24	558.5±31.27/550.8±25.09	N/A	N/A	45.30±7.97/40±5.39	44.55±8.11/43.95±5.85	1	8
Weigang Zeng, 2010	54	32	N/A	N/A	N/A	N/A	N/A	N/A	0	2
Weigang Zeng, 2007	118	108	N/A	N/A	N/A	N/A	N/A	N/A	0	60
Jiang Wu, 2015	33	30	528.46±78.42/436.14±83.27	521.47±80.12/446.72±67.46	26.24±12.08/10.37±2.58	27.18±11.83/12.43±3.19	40.72±13.06/16.87±2.26	39.24±13.27/24.35±4.03	2	6
Jing Chen, 2017	58	47	542.15±36.79/384.20±47.28	538.76±42.53/437.21±42.13	N/A	N/A	N/A	N/A	N/A	N/A
Minghai Zhou, 2014	19	15	N/A	N/A	N/A	N/A	N/A	N/A	0	3
Guangheng Cui, 2011	55	56	N/A	N/A	N/A	N/A	N/A	N/A	N/A	N/A
Yangang Wang, 2005	N/A	N/A	N/A	N/A	N/A	N/A	N/A	N/A	N/A	N/A
Hai Lu, 2013	29	25	564±80.5/326±20.3	518±75.4/349±34.7	36.1±4.3/14.6±5.6	34.2±6.8/18.4±3.9	65.7±18.5/22.6±7.3	63.4±11.7/29.6±6.9	2	1
Bo Duan, 2016	47	46	673.31±25.26/421.43±58.90	666.42±31.54/432.27±70.11	36.83±9.62/15.42±8.94	34.56±10.11/17.02±7.78	20.52±9.98/8.23±5.83	18.95±10.01/8.99±6.37	19	24
Guoying Han, 2010	39	29	568.6±92.5/412.5±94.7	581.7±102.4/483.4±107.6	N/A	N/A	N/A	N/A	N/A	N/A
Min Dai, 2015	36	29	N/A	N/A	N/A	N/A	N/A	N/A	N/A	N/A
Xiaohong He, 2008	27	20	508.0±63.6/276.1±23.2v	512.2±57.6/408.3±30.9	N/A	N/A	56.5±10.2/17.4±5.7	54.9±11.5/30.6±8.3	N/A	N/A
Xiaoxia Wang, 2010	28	29	N/A	N/A	N/A	N/A	N/A	N/A	N/A	N/A
Huijuan Yao, 2010	51	52	N/A	N/A	N/A	N/A	N/A	N/A	N/A	N/A
Lei Zhang, 2016	33	31	546.69±34.23/393.02±62.69	542.43±43.65/479.81±71.81	N/A	N/A	28.59±6.69/17.54±4.74	29.59±5.59/23.22±6.08	0	0
Bo Shi, 2010	38	25	N/A	N/A	N/A	N/A	N/A	N/A	N/A	N/A
Liping Yan, 2012	35	23	573.4±113.6/369.1±117.2	587.5±109.7/421.8±121.3	N/A	N/A	N/A	N/A	N/A	N/A
Yulei Wu, 2015	38	32	573.7±102.9/428.5±122.5	582.7±112.5/368.4±117.8	N/A	N/A	N/A	N/A	N/A	N/A
Yueqi Wang, 2013	30	29	542.36±54.55/351.12±43.92	534.99±58.81/424.73±56.79	30.90±10.15/6.70±5.11	31.45±10.75/12.65±5.26	28.90±6.53/6.74±3.14	29.45±11.58/7.67±4.11	0	7
Rong Li, 2014	69	62	525.68±114.85/308.30±50.96	537.01±126.46/375.60±61.17	N/A	N/A	N/A	N/A	N/A	N/A
Shanshan Yu, 2008	38	34	552.80±92.76/284.43±58.39	560.48±89.37/363.22±63.21	N/A	N/A	N/A	N/A	1	11
Weidong Qian, 2007	53	34	N/A	N/A	N/A	N/A	N/A	N/A	1	5
Zheng Huang, 2013	28	27	477.16±97.33/430.65±88.97	465.52±90.13/451.91±89.48	N/A	N/A	N/A	N/A	N/A	N/A
Chongyu Tan, 2013	48	34	568.73±38.29/326.81±38.14	573.77±39.87/371.90±38.39	N/A	N/A	N/A	N/A	4	16
Chun Kang, 2012	28	24	485.65±53.11/303.22±46.35	489.18±52.61/396.43±41.91	N/A	N/A	N/A	N/A	1	9
Xiaowu Fan, 2013	48	38	N/A	N/A	N/A	N/A	N/A	N/A	N/A	N/A
Jianchong Shen, 2014	57	49	495.99±50.73/312.78±54.03	485.50±47.58/378.07±50.89	N/A	N/A	N/A	N/A	1	5
Guiqiong Huang, 2016	55	38	547.48±50.93/304.28±60.14	546.37±46.65/415.96±51.86	26.17±10.28/5.98±1.45	25.94±9.45/16.05±1.76	35.18±8.82/15.79±4.21	34.95±7.88/23.57±4.52	N/A	17
Xin Ouyang, 2004	28	25	492.54±76.03/287.53±93.04	498.00±83.46/367.53±93.81	N/A	N/A	54.65±11.78/23.43±16.71	53.10±10.82/32.03±17.42	0	18
Zhimin Qi, 2016	101	93	515.13±118.04/318.57±76.12	508.67±121.38/392.06±78.39	24.60±7.68/8.82±2.58	23.86±7.44/12.26±2.71	48.91±12.68/17.58±5.22	48.36±12.27/22.14±6.15	4	16
Yaping Chen, 2014	40	37	N/A	N/A	N/A	N/A	N/A	N/A	15	32
Demei Li, 2012	38	34	496±66.9/266±48.6	498±49.6/562±51.6	35.4±6.5/15.6±5.2	38.5±7.8/21.9±6.1	68.3±15.6/21.5±12.5	64.9±12.6/30.2±7.9	5	16
Yanzhi Lin, 2009	28	22	506.57±119.90/352.41±79.93	548.70±83.98/402.42±85.53	8.15±1.25/4.64±0.97	8.52±1.23/6.32±1.09	32.62±13.82/17.9±5.2	32.40±13.93/26.4±10.87	N/A	17
Dehe Li, 2007	36	26	N/A	N/A	N/A	N/A	N/A	N/A	N/A	N/A
Jun Hao, 2014	29	23	N/A	N/A	N/A	N/A	N/A	N/A	0	11
Chongqing Yang, 2006	27	28	487.27±98.87/404.88±95.48	N/A	N/A	N/A	N/A	N/A	N/A	N/A
Lin Wang, 2011	26	27	498.33±87.25/491.13±86.89	498.28±128.18/482.23±116.51	29.94±22.06/9.24±5.7	52.46±56.82/18.16±28.10	N/A	N/A	0	1
Ayijiaman, 2012	54	37	539.16±34.49/376.3±52.85	552.29±46.15/425.79±48.69	N/A	N/A	N/A	N/A	N/A	N/A
Chifeng Wang, 2014	33	30	482.5±51.3/388.3±50.4	481.5±50.9/420.2±55.9	24.4±3.6/15±2.6	24.34±3.7/16±2.9	26.3±4.3/13.6±7.2	26.2±4.5/15.8±6.8	N/A	N/A
Jianping Luo, 2010	N/A	N/A	545.1±5.1/501.2±5.2	546.9±4.9/518.1±5	N/A	N/A	N/A	N/A	N/A	N/A
Xianzhang Zeng, 2013	58	51	589.35±92.87/317.26±89.23	578.34±95.31/323.44±92.59	N/A	N/A	N/A	N/A	N/A	N/A
Jinjin Jia, 2010	26	23	488.3±78.4/285.75±75.6	490.2±72.5/433.5±77.5	N/A	N/A	N/A	N/A	0	5
Xiaozhong Yu, 2013	31	28	556±23.1/216.12.6	552.±21.8/221±15.2	N/A	N/A	N/A	N/A	1	15
Jinfeng Li, 2013	34	33	468.39±100.28/342.47±90.26	452.28±98.75/437.65±94.57	N/A	N/A	N/A	N/A	N/A	N/A

^a^EG: experimental group, CG: control group;  ^b^SUA: serum uric acid;  ^c^CRP: C-reactive protein;  ^d^ESR: Erythrocyte Sedimentation Rate;  ^e^N/A: not applicable.
